# A case of cirrhosis with gastric varices and hypersplenism treated by endoscopic ultrasound-guided devascularization and partial splenic embolization (with video)

**DOI:** 10.1055/a-2749-8056

**Published:** 2025-12-19

**Authors:** Tianze Shi, Jialiang Huang, Guilian Cheng, Duanmin Hu

**Affiliations:** 1 105860 Department of Gastroenterology, Second Affiliated Hospital of Soochow University, Suzhou, China

Variceal bleeding and hypersplenism are major complications of cirrhosis. While endoscopic *N* -butyl-2-cyanoacrylate injection and partial splenic embolization (PSE) are recommended 1 , they entail risks of pulmonary embolism and splenic abscess. Recently, endoscopic ultrasound (EUS)-guided vascular intervention has emerged as an alternative 2 . In this case, we combined EUS-guided selective variceal devascularization (EUS-SVD) and EUS-guided PSE (EUS-PSE) to manage cirrhosis-induced gastric varices and hypersplenism.

A 61-year-old man with alcoholic cirrhosis was referred to our hospital for recurrent melena. Despite previous transfusion, he was experiencing severe anemia due to hypersplenism. Endoscopy revealed esophagogastric varices and a venous aneurysm on the posterior gastric wall ( Fig. 1 **a, b** ). Based on multidisciplinary assessment, we opted for EUS-SVD and EUS-PSE ( Video 1 ). During the EUS-SVD procedure, the left gastric vein was identified and occluded using sequential sclerotherapy with lauromacrogol/tissue adhesive and an 8 × 12mm coil deployment ( Fig. 1 **c** ). Successful thrombosis was confirmed, evidenced by a hyperechoic shadow and the absence of blood flow ( Fig. 1 **d** ). After 1 month without rebleeding or adverse events, EUS-guided PSE was performed. Under EUS guidance, a primary splenic artery branch was identified and punctured with a 22G needle. An 8 mm × 12 mm coil was deployed, followed by lauromacrogol for tracing and 1 mL tissue adhesive for embolization ( Fig. 2 **a** ). Subsequent imaging identified a lack of flow signal in the splenic artery and hyperechoic nodular in splenic parenchyma, indicating ischemia ( Fig. 2 **b** ). Postoperatively, the patient experienced a transient fever of 38.6°C, managed with imipenem for 7 days. CT on day 4 confirmed over 50% splenic infarction and proper coil placement ( Fig. 2 **c, d** ). The patient’s stool color normalized by discharge, without rebleeding or adverse events during 1-month follow-up.

Endoscopic ultrasound-guided devascularization for gastric varices and endoscopic ultrasound-guided partial splenic embolization for hypersplenism.Video 1

**Fig. 1:**
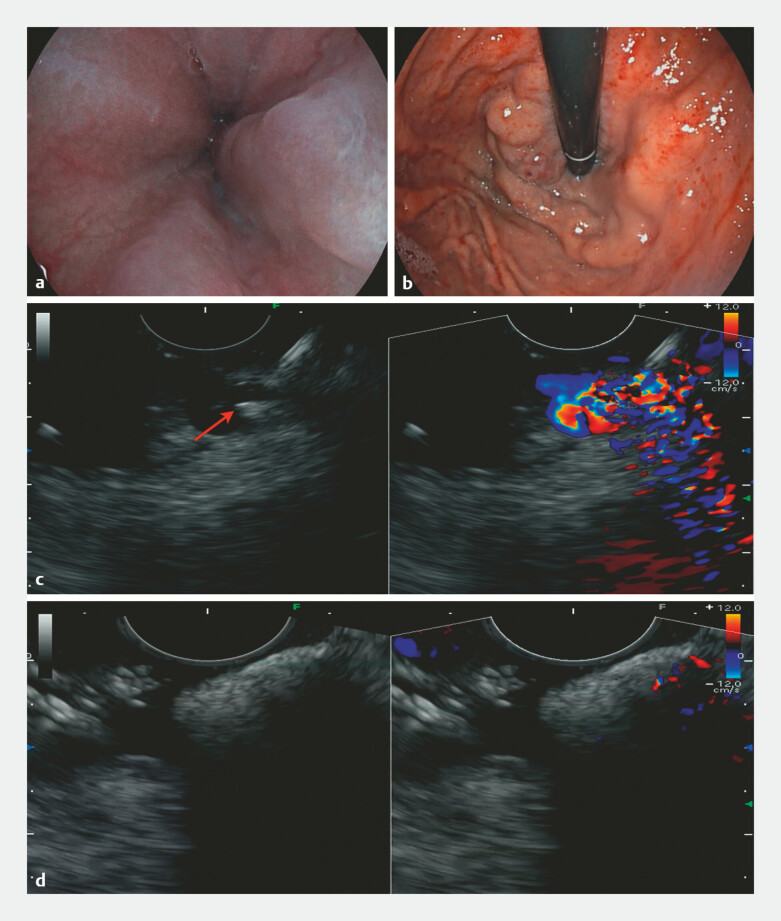
EUS-SVD in patient with gastric varices. **a, b** Endoscopy identified varices at the end of the esophagus (red color sign−) and venous aneurysm at the fundus of the stomach (red color sign+). **c** During lauromacrogol injection, intravascular hyperechoic shadows (red arrow) confirmed the needle in the left gastric vein. **d** After embolization, the blood flow signal in the venous cluster disappeared, replaced by the hyperechoic shadow. EUS-SVD, EUS-guided selective variceal devascularization.

**Fig. 2:**
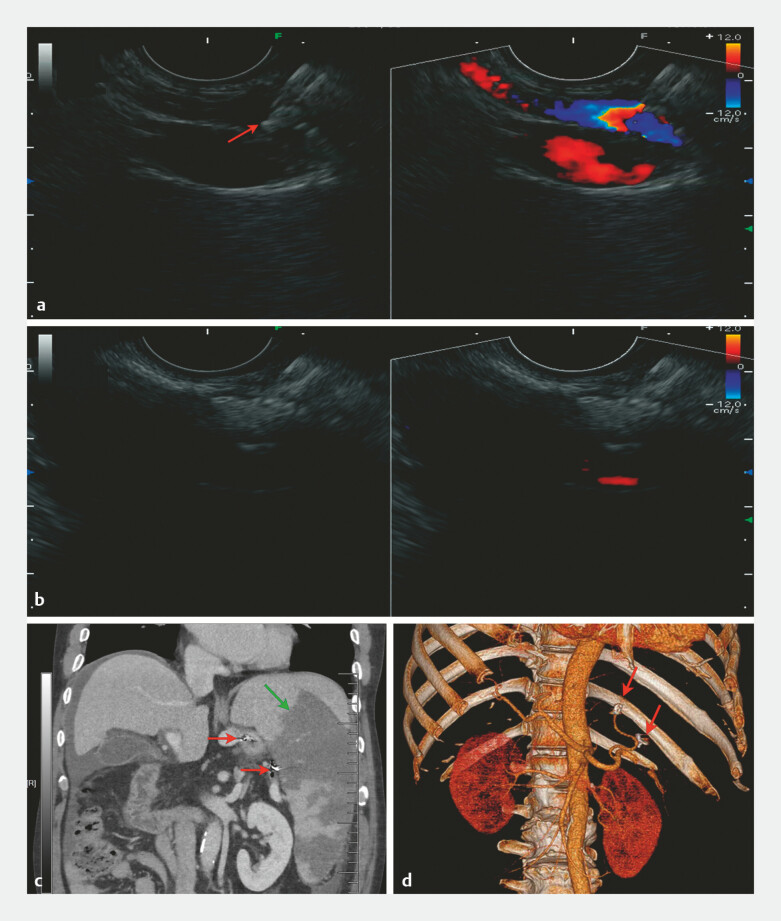
EUS-PSE for the treatment of hypersplenism. **a** Under EUS guidance, an 8 mm × 12 mm coil was placed in the splenic artery (red arrow). **b** EUS identified the disappearance of the blood flow signal in the primary splenic artery branch after embolization. **c** CT revealed over 50% spleen infarction (green arrow) and the embolization coils (red arrow). **d** Three-dimensional CT reconstruction showed embolization coils in the left gastric vein and primary splenic artery branch (red arrow). CT, computed tomography; EUS, endoscopic ultrasound; EUS-PSE, EUS-guided partial splenic embolization.

EUS-SVD demonstrates 95% clinical success with under 15% adverse events. No adverse events have been reported with EUS-PSE 3 . Herein, the combined therapy achieved success for cirrhosis-induced gastric varices and hypersplenism. Postoperative fever was attributed to sterile inflammation and absorption, which resolved in 3 days without CT evidence of splenic abscess.

Endoscopy_UCTN_Code_TTT_1AS_2AG

## Notes

Endoscopy E-Videos https://eref.thieme.de/e-videos
*E-Videos* is an open access online section of the journal *Endoscopy* , reporting on interesting cases and new techniques in gastroenterological endoscopy. All papers include a high-quality video and are published with a Creative Commons CC-BY license. Endoscopy E-Videos qualify for HINARI discounts and waivers and eligibility is automatically checked during the submission process. We grant 100% waivers to articles whose corresponding authors are based in Group A countries and 50% waivers to those who are based in Group B countries as classified by Research4Life (see: https://www.research4life.org/access/eligibility/ ). This section has its own submission website at https://mc.manuscriptcentral.com/e-videos .
